# A comprehensive investigation of variants in genes encoding adiponectin (*ADIPOQ*) and its receptors (*ADIPOR1/R2*), and their association with serum adiponectin, type 2 diabetes, insulin resistance and the metabolic syndrome

**DOI:** 10.1186/1471-2350-14-15

**Published:** 2013-01-25

**Authors:** Kirsten E Peters, John Beilby, Gemma Cadby, Nicole M Warrington, David G Bruce, Wendy A Davis, Timothy ME Davis, Steven Wiltshire, Matthew Knuiman, Brendan M McQuillan, Lyle J Palmer, Peter L Thompson, Joseph Hung

**Affiliations:** 1 School of Medicine and Pharmacology, Fremantle Hospital Unit, The University of Western Australia, Nedlands, Western Australia, Australia; 2 Department of Diagnostic Molecular Genomics, PathWest, Queen Elizabeth II Medical Centre, Nedlands, Nedlands, Western Australia, Australia; 3 School of Pathology and Laboratory Medicine, The University of Western Australia, Nedlands, Western Australia, Australia; 4 Centre for Genetic Origins of Health and Disease, University of Western, Nedlands, Western Australia, Australia; 5 School of Women’s and Infants’ Health, University of Western Australia, Nedlands, Western Australia, Australia; 6 Centre for Medical Research, Western Australian Institute for Medical Research, The University of Western Australia, Nedlands, Australia; 7 School of Population Health, The University of Western Australia, Nedlands, Western Australia, Australia; 8 School of Medicine and Pharmacology, Sir Charles Gairdner Hospital Unit, University of Western Australia, Nedlands, Western Australia, Australia; 9 Genetic Epidemiology and Biostatistics Platform, Ontario institute for Cancer Research, Toronto, Canada

**Keywords:** Adiponectin, *ADIPOQ*, *ADIPOR*, Type 2 diabetes, Insulin resistance and Metabolic syndrome

## Abstract

**Background:**

Low levels of serum adiponectin have been linked to central obesity, insulin resistance, metabolic syndrome, and type 2 diabetes. Variants in *ADIPOQ*, the gene encoding adiponectin, have been shown to influence serum adiponectin concentration, and along with variants in the adiponectin receptors (*ADIPOR1* and *ADIPOR2*) have been implicated in metabolic syndrome and type 2 diabetes. This study aimed to comprehensively investigate the association of common variants in *ADIPOQ, ADIPOR1* and *ADIPOR2* with serum adiponectin and insulin resistance syndromes in a large cohort of European-Australian individuals.

**Methods:**

Sixty-four tagging single nucleotide polymorphisms in *ADIPOQ*, *ADIPOR1* and *ADIPOR2* were genotyped in two general population cohorts consisting of 2,355 subjects, and one cohort of 967 subjects with type 2 diabetes. The association of tagSNPs with outcomes were evaluated using linear or logistic modelling. Meta-analysis of the three cohorts was performed by random-effects modelling.

**Results:**

Meta-analysis revealed nine genotyped tagSNPs in *ADIPOQ* significantly associated with serum adiponectin across all cohorts after adjustment for age, gender and BMI, including rs10937273, rs12637534, rs1648707, rs16861209, rs822395, rs17366568, rs3774261, rs6444175 and rs17373414. The results of haplotype-based analyses were also consistent. Overall, the variants in the *ADIPOQ* gene explained <5% of the variance in serum adiponectin concentration. None of the *ADIPOR1/R2* tagSNPs were associated with serum adiponectin. There was no association between any of the genetic variants and insulin resistance or metabolic syndrome. A multi-SNP genotypic risk score for *ADIPOQ* alleles revealed an association with 3 independent SNPs, rs12637534, rs16861209, rs17366568 and type 2 diabetes after adjusting for adiponectin levels (OR=0.86, 95% CI=(0.75, 0.99), P=0.0134).

**Conclusions:**

Genetic variation in *ADIPOQ*, but not its receptors, was associated with altered serum adiponectin. However, genetic variation in *ADIPOQ* and its receptors does not appear to contribute to the risk of insulin resistance or metabolic syndrome but did for type 2 diabetes in a European-Australian population.

## Background

Adiponectin is the most abundant adipose tissue-derived cytokine. It has anti-inflammatory, anti-diabetic and anti-atherogenic properties, and low circulating levels are associated with central obesity, insulin resistance, metabolic syndrome (MetS), and type 2 diabetes (T2D) [1].

Serum adiponectin concentrations are highly heritable, and a number of genome-wide association studies (GWAS) have identified *ADIPOQ,* the gene encoding adiponectin, as the main locus contributing to variations in serum levels in European and Asian populations [2-5]. Cross-sectional studies in healthy and diabetic populations have provided further evidence for the association of single nucleotide polymorphisms (SNPs) in *ADIPOQ* with serum adiponectin concentrations [6-10]. Several studies have linked *ADIPOQ* variants to T2D and MetS, although the results to date have been discordant and not replicated across whole populations [5,8-12]. Nevertheless, examining the genes which affect serum adiponectin levels may help to confirm adiponectin as a cause or consequence of MetS and T2D using a Mendelian randomisation approach [13].

Adiponectin cellular signalling is mediated by two adiponectin receptors. The genes for these (*ADIPOR1* and *ADIPOR2*), although generally not associated with serum adiponectin, have themselves been implicated in insulin resistance and T2D risk in genetic association studies, but also with inconsistent results [14-18].

In light of this background, the aims of the present study were i) to examine the evidence for association between total serum adiponectin levels and variants in *ADIPOQ* and *ADIPOR1/R2* using a comprehensive tagSNP approach and haplotype-based analysis, ii) to assess associations between SNPs in these genes and total serum adiponectin concentrations in large, well-characterised and independent Western Australian cohorts (two general population and one adult T2D cohort), and iii) to investigate the relationship between gene variants found to be associated with adiponectin levels and relevant clinical outcomes including MetS and T2D.

## Methods

### Subjects

This study examined subjects from three Western Australian cohorts: the Busselton Population Health Survey (BHS) [19], the Carotid Ultrasound Disease Assessment Study (CUDAS) [20] and the Fremantle Diabetes Study (FDS) [21].

The BHS and CUDAS cohorts are representative of the general population and do not contain individuals with T2D, while FDS contains individuals with T2D who are representative of people with diabetes in an urban Australian setting. These cohorts are predominantly European-Australian. The present study was restricted to individuals who had both serum adiponectin and genotyping data available.

The subset of the BHS cohort with available data consisted of 1,322 unrelated adult individuals who were recruited as part of a larger cross-sectional community study in 1994/1995 [22]. The CUDAS cohort comprised 1,033 individuals selected from a random electoral survey from the Perth metropolitan area [20]. The FDS cohort of 967 diabetic individuals was recruited from a longitudinal observational study [21]. All study participants gave written informed consent, and the study protocol was approved by the University of Western Australia Human Research Ethics Committee, the Busselton Population Medical Research Foundation, and the Human Rights Committee at Fremantle Hospital.

### Biochemical and anthropomorphic analyses

A fasting blood sample was obtained from each subject. Total serum adiponectin levels were measured by a commercially available quantitative sandwich enzyme immunoassay technique (R&D Systems Inc., Minneapolis, Minnesota, USA). The intra-assay coefficients of variation ranged from 2.1 to 4.3% and the inter-assay coefficients of variation ranged from 5.4 to 9.6%. Plasma glucose, insulin, total cholesterol, LDL- and HDL-cholesterol, and triglycerides were determined by standard methods [19-21]. Insulin resistance (IR) was determined using the homeostatic model of assessment scores (lnHOMA-IR) [23]. Anthropometric measurements and resting blood pressure were taken according to standard clinical procedures. Body mass index (BMI) was calculated as weight (kg)/height (m)^2^. Metabolic syndrome (MetS) was defined according to the recently updated National Cholesterol Education Program (NCEP) criteria [24]. Individuals with three or more of the following criteria were classified as having MetS: increased waist circumference (≥102 cm in men, ≥88 cm in women), hypertriglyceridaemia ≥1.70 mmol/L (≥150 mg/dL), low HDL-cholesterol <1.00 mmol/L in men and <1.3 mmol/L in women (<40 mg/dL and <50 mg/dL), high blood pressure (systolic ≥130 and/or diastolic ≥85 mm Hg, or current use of antihypertensive therapy) and high fasting glucose ≥5.6 mmol/L (≥100 mg/dL). Data for clinical and demographic characteristics (Table 1) are given as proportions, mean±SD, or geometric mean (SD range).

**Table 1:** Baseline clinical, metabolic, and demographic characteristics of each cohort by gender

	**BHS**		**CUDAS**		**FDS**	
	**Female (n=736)**	**Male (n=586)**	**Female (n=521)**	**Male (n=512)**	**Female (n=489)**	**Male (n=478)**
Adiponectin (mg/L)	14.2 (8.1–24.8)	7.9 (4.2–15.0)	13.9 (8.1–23.8)	7.9 (4.5–14.0)	8.6 (4.4–16.6)	5.9 (3.0–11.4)
Age (years)	51.3±17.8	51.5±17.7	52.8±12.7	53.2±12.8	64.6±11.4	64.4±10.3
SBP (mm Hg)	122±20	126±16	126±20	129±16	150±23	151±23
DBP (mm Hg)	73±10	77±10	78±11	82±9	79±11	83±11
Fasting Glucose (mmol/L)	4.8±0.5	4.9±0.5	5.3±0.7	5.3±0.8	8.7 (6.3–12.0)	8.6 (6.2–11.8)
Fasting Insulin (IU/L)*	41.6 (24.6–70.3)	44.6 (24.6–80.8)	33.6 (18.4–61.4)	36.1 (19.1–68.5)	80.7 (44.2–147.2)	68.0 (34.6–133.4)
lnHOMA-IR (insulin resistance)	0.9 (0.5–1.5)	0.9 (0.5–1.7)	0.6 (0.3–1.2)	0.7 (0.4–1.3)	1.7 (0.9–3.2)	1.4 (0.7–2.8)
Total cholesterol (mmol/L)	5.6±1.2	5.5±1.0	5.6±1.0	5.5±1.0	5.7±1.1	5.2±1.1
LDL-cholesterol (mmol/L)	3.5±1.0	3.7±0.9	3.6±0.9	3.7±0.9	3.6±1.0	3.3±0.8
HDL-cholesterol (mmol/L)	1.51 (1.17–1.95)	1.18 (0.91–1.54)	1.47 (1.14–1.88)	1.14 (0.89–1.47)	1.09 (0.82–1.46)	0.93 (0.68–1.26)
Serum triglycerides (mmol/L)	1.0 (0.6–1.7)	1.2 (0.7–2.2)	1.0 (0.6–1.6)	1.2 (0.7–2.1)	1.9 (1.2–3.0)	1.9 (1.0–3.4)
Body Mass Index (BMI) (kg/m^2^)	25.1±4.4	26.3±3.5	25.3±4.4	26.6±3.5	30.2±5.9	28.9±4.5
Waist circumference (cm)	79.7±11.0	92.9±10.2	76.9±10.4	92.0±9.2	96.4±12.6	103.4±11.2
Waist-Hip ratio	0.79±0.06	0.93±0.06	0.76±0.06	0.90±0.05	0.86±0.06	0.96±0.06
Antihypertensive tx (%)	19.2	16.1	13.8	13.9	56.4	45.4
Smoking (never/ex/current) (%)	60.2/28.9/10.9	47.6/37.1/15.2	62.8/24.2/13.1	39.1/43.6/17.4	64.4/23.6/12.0	24.9/57.4/17.6
NCEP_Hypertension (%)	40.0	46.2	47.0	54.7	92.8	91.0
NCEP_Hyperglycaemia (%)	7.1	10.0	33.9	40.2	94.3	93.5
NCEP_Hypertriglyceridemia (%)	17.4	28.9	14.8	30.7	59.0	58.7
NCEP_LowHDL (%)	27.3	26.8	30.1	29.3	73.1	60.0
NCEP_Obese (%)	20.7	18.1	16.3	14.8	74.1	55.0
NCEP_MetSScore^†^ (%)						
0	39.8	31.7	27.2	18.6	0.4	0.2
1	29.9	32.3	29.5	27.1	1.0	2.0
2	16.6	18.3	26.4	29.6	8.5	15.8
3+	13.7	17.7	17.0	24.7	90.0	82.0

* insulin in non-insulin users ^†^ NCEP_MetSScore is the number of MetS components.

The data for continuous variables is mean±standard deviation, or geometic mean (standard deviation range). The data for categorical variables is shown as proportions.

### Selection and genotyping of tSNPs

A tagSNP (tSNP) approach (*r*^2^≥0.80, minor allele frequency ≥5%) was used to explore the genetic variation in *ADIPOQ, ADIPOR1,* and *ADIPOR2.* tSNPs were selected using Haploview to represent the common genetic variation of each gene, including an additional 10 kb upstream and downstream, as well as variants previously reported in the literature to be associated with serum adiponectin levels. Sixty-four tSNPs in *ADIPOQ* (8), *ADIPOR1* and *ADIPOR2* (15, 16) were selected and genotyped based on this approach (Additional file 1). Genotyping was performed on an Illumina BeadStation using the GoldenGate technology. DNA samples from CEPH trios (obtained from the Coriell Cell Repository) served as internal controls for quality of clustering and reproducibility. A random (10%) sample was analysed in duplicate, with reproducibility found to be 100% for the Busselton population and 99.9% for both CUDAS and FDS populations. Individual SNP concordance rates were between 99.3 and 100%. Deviations from Hardy–Weinberg equilibrium (HWE) (P<0.05) were examined for each tSNP using Fisher’s exact test, and minor allele frequencies determined for each cohort.

### Analysis of association between tSNPs and adiponectin levels

Adiponectin levels were normalised using a natural logarithm transformation prior to analysis. Associations between transformed values and genotypes at each tSNP were examined using a generalised linear model approach implemented in SimHap [25]. Each polymorphism was modelled as a genotypic (codominant) genetic effect, accommodating the effects of age, gender, and BMI as significant covariates. We determined marginal geometric mean values of adiponectin according to genotype at each tSNP using SimHap. We corrected for the multiple testing inherent in this study using the false discovery rate method, and provide q-values [26] (Additional file 2). Statistical significance was defined at α<0.05.

We used Haploview [27] to determine the linkage disequilibrium (LD) between the selected tSNPs and characterise the haplotype block structure. Haplotypes were inferred for individuals with ambiguous phase and haplotype frequencies were estimated using an expectation–maximization algorithm as implemented in SimHap. Haplotypes were recorded as independent factors into three classes (0, 1, or 2), representing the number of copies of each haplotype in an individual’s diplotype. The effect of each individual haplotype was calculated relative to not having that haplotype. To investigate associations with serum adiponectin, common haplotypes (frequency >5%) were examined using a codominant model adjusted for age, gender and BMI (Additional file 3).

Meta-analysis of the *ADIPOQ* polymorphisms against total serum adiponectin in the three cohorts was carried out using the rmeta package in R version 2.8.0. All three cohorts were pooled after checking for genetic heterogeneity for each tSNP. Random-effects modelling were used for the meta-analysis as we assumed that the cohorts would be heterogeneous.

### Analysis of association between tSNPs and T2D, lnHOMA-IR and MetS

Associations between tSNPs and T2D, log transformed HOMA-IR (lnHOMA-IR) and MetS were evaluated in SimHap using a codominant model corrected for age, gender and BMI. For T2D, the FDS cohort was compared to control individuals without a history of diabetes from the combined BHS and CUDAS cohorts. LnHOMA-IR was modelled as a quantitative outcome, while MetS was analysed as an ordinal outcome in the BHS and CUDAS cohorts for the number of MetS criteria met. In addition to investigating the tSNPs independently, an allele score was created by summing the number of risk alleles an individual had in those tSNPs that were shown to be associated with adiponectin levels in the meta-analysis.

### Power analysis

A power calculation, performed in the Quanto software [28], indicated that this study has at least 90% power at an alpha level of p=0.05 to detect an odds ratio for diabetes (population risk of 10%) of 1.45 for a minor allele frequency of 0.05 and an odds ratio of 1.2 for a minor allele frequency of 0.45, under an additive genetic model.

## Results

### Sample characteristics

A total of 3,322 subjects from the three cohorts were included (Table 1). As expected, the two general population-based cohorts differed significantly to the diabetic cohort in cardiometabolic risk characteristics, reflecting the different recruitment criteria. The BHS and CUDAS populations were similar with regards serum adiponectin concentrations, age and BMI. Compared with the BHS and CUDAS cohorts, the FDS population was older, had lower serum adiponectin concentrations, and had higher BMI and systolic blood pressure. In all three cohorts, females had higher serum adiponectin concentrations than males.

### Genotype distribution and linkage disequilibrium

Of the sixty-four genotyped tSNPs, five failed and six were monomorphic, leaving fifty-three that were included in the association analyses (21 in *ADIPOQ*, 13 in *ADIPOR1*, and 19 in *ADIPOR2*). Details of these tSNPs including genotype and allele frequencies are reported in Additional file 1. Allele frequencies for all tSNPs were similar to those reported in HapMap (http://hapmap.ncbi.nlm.nih.gov/). The genotype distribution of each tSNP was consistent with HWE in all populations, with the following exceptions: two tSNPs in BHS (*ADIPOR1* rs75114693 and *ADIPOR1* rs6666089) and one tSNP in CUDAS (*ADIPOR1* rs6666089) (all *P*<0.05).

**Figure 1:**
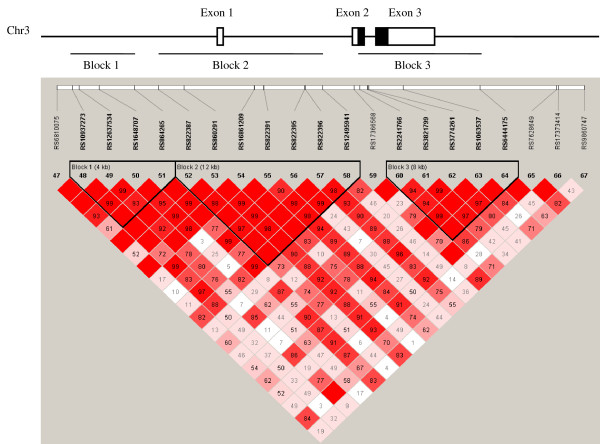
**Linkage disequilibrium plot of SNPs within *ADIPOQ *using data from the combined BHS/CUDAS cohorts.** Schematic representation of the adiponectin (*ADIPOQ*) gene on chromosome 3. The exon/intron structure of the gene is shown, together with the pairwise linkage disequilibrium (LD) and haplotype blocks across the gene. The protein coding region is shown by black shading in exons 2 and 3. The D’ values for each SNP pair are shown, with dark red diamonds without a corresponding D’ value representing complete LD (D’=1).

Analysis of pairwise LD in *ADIPOQ* showed three LD blocks (Figure 1), which were similar between all three cohorts. Haplotypes were analysed within each block for association with the outcomes of interest. A strong correlation (D’>0.90 and r^2^>0.8) was found between tSNPs rs6810075 and rs1648707, rs864265 and rs860291, rs822387 and rs16861209, rs822391 and rs822396, and rs2241766 and rs1063537 within each cohort. Statistical analysis showed that the effects of each of these tSNP pairs on adiponectin levels were identical, and for this reason we chose to report on only one tSNP from each pair: rs1648707, rs860291, rs16861209, rs822396 and rs1063537. There was no interaction between gender and any of the genetic variants, so males and females were combined for all subsequent analyses.

### Association of single tSNPs with serum adiponectin

Age, gender and BMI accounted for 35.5% of the variation in serum adiponectin in the BHS cohort, 33.2% in CUDAS, and 22.0% in FDS. After adjustment for these covariates and, under a codominant model, eight tSNPs in *ADIPOQ* were significantly associated with serum adiponectin in the BHS cohort: rs12637534, rs1648707, rs16861209, rs17366568, rs3774261, rs1063537, rs6444175 and rs17373414 (all q<0.05). Of these, four were significant in CUDAS (rs1648707, rs16861209, rs3774261, and rs6444175), and two in FDS (rs16861209 and rs17366568). Full results (for these eight SNPs) for each cohort, including the data for tSNPs in LD, are shown in Additional file 2. None of the tSNPs in *ADIPOR1/R2* were associated with serum adiponectin in any of the cohorts.

After adjustment for age, gender and BMI, tSNPs rs12637534, rs16861209 and rs17366568 accounted for 4.8% of the variance in serum adiponectin in BHS, rs1648707 and rs16861209 explained 2.9% of the variance in CUDAS, and rs16861209 and rs17366568 accounted for 1.7% in FDS. The minor allele at rs16861209 (A allele) was consistently associated with increased serum adiponectin across all three cohorts, specifically 3.7 mg/L in BHS, 2.7 mg/L in CUDAS and 1.3 mg/L in FDS (Additional file 2).

Meta-analysis of all *ADIPOQ* tSNPs in our three cohorts is shown in Table 2. tSNPs in LD gave identical results so only data for one tSNP from each pair are presented. As FDS displayed different mean levels of adiponectin and other quantative traits, a second meta-analysis was conducted without the FDS study and similar results were found (data not shown).

**Table 2:** **Meta-analysis of the association between *ADIPOQ *SNPs and serum adiponectin in our combined cohort of 3,431 individuals**

**SNP**	**Number of individuals**	**β (95% CI)**	**P-value**	**P-value (heterogeneity)**
rs10937273				
GA	1576	0.0004 (−0.041, 0.041)	0.98	0.63
AA	577	0.081 (0.027, 0.134)	3.30E-03	0.83
rs12637534				
AG	481	−0.147 (−0.209, -0.085)	3.39E-06	0.26
GG	18	0.076 (−0.171, 0.323)	0.55	0.63
rs1648707				
AC	1473	−0.090 (−0.129, -0.050)	8.06E-06	0.44
CC	392	−0.192 (−0.252, -0.131)	5.41E-10	0.80
rs860291				
CT	767	0.028 (−0.017, 0.072)	0.22	0.61
TT	50	0.089 (−0.064, 0.243)	0.25	0.97
rs16861209				
CA	530	0.246 (0.179, 0.313)	8.14E-13	0.17
AA	27	0.337 (0.129, 0.545)	1.50E-03	0.61
rs822395				
AC	1465	0.052 (0.007, 0.098)	0.02	0.28
CC	404	0.069 (0.009, 0.129)	0.03	0.88
rs822396				
AG	998	0.002 (−0.039, 0.043)	0.92	0.90
GG	129	0.010 (−0.087, 0.108)	0.84	0.49
rs12495941				
GT	1500	0.006 (−0.034, 0.046)	0.77	0.41
TT	386	0.056 (−0.035, 0.148)	0.23	0.11
rs17366568				
GA	691	−0.146 (−0.205, -0.087)	1.22E-06	0.19
AA	41	−0.370 (−0.543, -0.197)	2.72E-05	0.34
rs3821799				
CT	1573	0.024 (−0.034, 0.082)	0.42	0.16
TT	685	0.051 (−0.002, 0.104)	0.06	0.52
rs3774261				
GA	1502	0.060 (0.019, 0.010)	3.80E-03	0.63
AA	517	0.176 (0.120, 0.231)	4.98E-10	0.60
rs1063537				
CT	659	0.026 (−0.045, 0.096)	0.48	0.11
TT	47	0.233 (−0.203, 0.669)	0.29	0.001
rs6444175				
GA	1300	0.067 (0.028, 0.106)	7.00E-04	0.45
AA	229	0.180 (0.105, 0.255)	2.40E-06	0.74
rs7628649				
CT	656	0.031 (−0.015, 0.077)	0.19	0.51
TT	47	0.188 (−0.082, 0.458)	0.17	0.05
rs17373414				
CT	654	0.116 (0.069, 0.163)	1.64E-06	0.35
TT	35	−0.004 (−0.186, 0.179)	0.97	0.39
rs9860747				
CT	871	0.018 (−0.024, 0.061)	0.40	0.67
TT	90	0.073 (−0.042, 0.187)	0.21	0.43

The meta-analysis showed a consistent, homogeneous effect of seven of the eight tSNPs associated with serum adiponectin in the BHS cohort (q<0.05). tSNP rs1063537 (and rs2241766 via strong LD) showed significant evidence of heterogeneity (*P*=0.001), indicating differences between the cohorts at this marker. Two additional tSNPs (rs10937273 and rs822395) were also associated with serum adiponectin in the meta-analysis of the three combined cohorts. Carriage of the minor alleles at rs12637534, rs1648707 and rs17366568 was associated with lower serum adiponectin levels (*P*<1×10^-5^), while the minor alleles at rs10937273, rs16861209, rs822395, rs3774261, rs6444175 and rs17373414 was associated with higher serum adiponectin levels (*P*≤0.02).

To determine which of the 9 SNPs identified in the meta-analysis independently contribute to adiponectin variation, we conducted a conditional regression analysis in all three cohorts; this analysis showed that 3 of the top 9 SNPs were independently associated with adiponectin levels in the BHS, including rs12637534, rs16861209 and rs17366568. Similar results were found in the other two studies, where both rs16861209 and rs17366568 remained significant in FDS, while rs16861209, rs17366568 and rs1648707 were significant in CUDAS.

### Associations of haplotypes with serum adiponectin

Associations between the most frequent haplotypes and serum adiponectin were analysed after correcting for age, gender and BMI in each cohort (data not shown). All cohorts were combined and meta-analysis performed on these haplotypes using random effects modelling (Table 3). The haplotype-based associations were consistent with the results from the single tSNP analyses.

**Table 3:** **Meta-analysis of the association between *ADIPOQ *haplotypes and serum adiponectin in our combined cohort of 3,431 individuals**

**Block**	**Haplotype and copy number**	**β (95% CI)**	**P-value**	**P-value (heterogeneity)**
1	AAAG			
	1	0.001 (−0.041, 0.043)	0.96	0.67
	2	0.081 (0.026, 0.135)	3.70E-03	0.74
	GACG			
	1	−0.071 (−0.110, -0.032)	4.00E-04	0.79
	2	−0.109 (−0.183, -0.035)	3.70E-03	0.61
	GAAT			
	1	−0.047 (−0.089, -0.005)	3.00E-02	0.62
	2	0.026 (−0.099, 0.152)	0.68	0.85
	GAAG			
	1	0.219 (0.142, 0.296)	2.55E-08	0.11
	2	0.280 (0.070, 0.490)	9.20E-03	0.89
	GGCG			
	1	−0.152 (−0.231, -0.092)	8.22E-07	0.28
	2	0.051 (−0.188, 0.291)	0.67	0.60
2	TCCTAAT			
	1	0.007 (−0.036, 0.049)	0.76	0.34
	2	0.052 (−0.048, 0.153)	0.31	0.08
	TCCTAAG			
	1	−0.069 (−0.108, 0.030)	6.00E-04	0.54
	2	−0.115 (−0.183, -0.046)	1.00E-03	0.64
	TTCCCGG			
	1	0.026 (−0.021, 0.073)	0.28	0.73
	2	0.122 (−0.035, 0.278)	0.13	0.96
	CCATCAG			
	1	0.244 (0.183, 0.305)	5.11E-15	0.29
	2	0.301 (0.079, 0.523)	7.80E-03	0.88
	TCCTCAG			
	1	−0.156 (−0.228, -0.084)	2.42E-05	0.22
	2	−0.402 (−0.066, -0.146)	2.10E-03	0.74
	TCCCCGG			
	1	−0.067 (−0.129, -0.006)	3.11E-02	0.37
	2	0.088 (−0.217, 0.393)	0.57	0.45
3	TCGCG			
	1	−0.030 (−0.081, 0.022)	0.25	0.34
	2	−0.044 (−0.096, 0.008)	0.09	0.44
	TTACA			
	1	0.071 (0.032, 0.111)	4.00E-04	0.57
	2	0.190 (0.114, 0.267)	1.01E-06	0.88
	GTATG			
	1	0.022 (−0.049, 0.094)	0.54	0.12
	2	0.220 (−0.207, 0.647)	0.31	0.001
	TTGCG			
	1	−0.232 (−0.297, -0.167)	2.87E-12	0.31
	2	−0.186 (−0.597, 0.225)	0.38	0.14

Block 1: rs10937273-rs12637534-rs1648707-rs864265.

Block 2: rs822387-rs860291-rs16861209-rs822391-rs822395-rs822396-rs12495941.

Block 3: rs2241766-rs3821799-rs3774261-rs1063537-rs6444175.

In block 1, five common haplotypes were observed that significantly affected serum adiponectin. Haplotypes carrying the major allele at rs12637534 and rs1648707 (A-A) were found to increase serum adiponectin (all *P≤*9.20×10^-3^), with the exception of the GAAT haplotype where one copy was associated with decreased serum adiponectin concentrations (*P*=0.03). The opposite was true for carriage of the minor allele at each of these tSNPs, with one copy of GGCG associated with a significantly decreased serum adiponectin (*P=*8.22×10^-7^). One haplotype, GACG, carrying the major allele at rs12637534 (A) and minor allele at rs1648707 (C), was also associated with a decreased serum adiponectin, suggesting that the effect of rs1648707 may be stronger than rs12637534. This seems likely given the lower minor allele frequency of rs12637534 compared to rs1648707 (8% vs 34%).

In block 2, the CCATCAG haplotype (frequency 8%) carrying the minor alleles at rs16861209 (A) and rs822395 (C) (both associated with a higher serum adiponectin) was found to be significantly associated with serum adiponectin (*P=*5.11×10^-15^). This haplotype was found to be significant in each cohort and explained 2.3% of the variation in levels in BHS, 2.0% in CUDAS and 0.7% in FDS. Conversely, three haplotypes carrying the major allele at rs16861209 (associated with a lower serum adiponectin) were found to be significantly associated with a decreased serum adiponectin (all *P≤*3.11×10^-2^).

Two haplotypes in block 3 were significantly associated with serum adiponectin in the meta-analysis, and in each individual cohort. The TTACA haplotype (frequency ~25%) was associated with increased levels and TTGCG (frequency ~6%) with decreased levels (*P*≤4×10^-4^ and *P*≤2.87×10^-12^, respectively). The TTACA haplotype carries the minor allele (A allele) at both rs3774261 and rs6444175 that was shown in the single tSNP analyses to be associated with increased serum adiponectin, while TTGCG carries the major allele at each of these tSNPs and has the opposite effect on levels, again showing consistency between single tSNP and haplotype analysis. This haplotype accounted for 2.3% of the variation in levels in BHS, 0.7% in CUDAS and 0.6% in FDS. The more common TTACA haplotype only accounted for between 0.3 and 0.7% of the variation in levels across all three cohorts.

In all haplotype blocks, the single SNPs tagging the block accounted for the same, or slightly more of the variation in adiponectin levels than the haplotype block itself (difference of between 0–0.8% between the tSNPs and the haplotypes).

### Association of tSNPs with T2D, lnHOMA-IR and MetS

No significant associations were detected between genetic variants in *ADIPOQ*, or *ADIPOR1/R2,* and lnHOMA-IR or MetS in any cohort after correction for age, gender and BMI. We also found no association between the *ADIPOQ* and *ADIPOR1/R2* genetic variants and T2D in the case:control analysis (results not shown).

There was a significant association between the allele score of the top nine SNPs and T2D in the case–control study without adjusting for adiponectin levels (OR=0.94, 95% CI=(0.91,0.98), P=0.0015) and this association strengthened after adjusting for adiponectin levels (OR=0.92, 95% CI=(0.89, 0.96), P=2.43×10^-5^). The allele score of the 3 independent SNPs; rs12637534, rs16861209, rs17366568, were significantly associated with T2D only after adjusting for adiponectin levels (OR=0.86, 95% CI=(0.75, 0.99), P=0.0134).

## Discussion

The present study reports associations between total serum adiponectin concentrations and a range of genetic variations and haplotypes in the adiponectin gene (*ADIPOQ*) and genes for adiponectin receptors 1 and 2 *(ADIPOR1/R2*) in three independent European-Australian population-based cohorts. Several genetic variants in *ADIPOQ*, but not *ADIPOR1/R2*, were associated with serum adiponectin. These associations were consistent across the three cohorts and showed similar results for tSNP and haplotype-based analysis. After adjusting for age, gender, and BMI, variants in the *ADIPOQ* gene accounted for only 3.9% of the variation in serum adiponectin in the combined general population cohorts and 1.7% in the diabetic sample. There was also no association between tSNPs or haplotypes of *ADIPOQ,* and *ADIPOR1/R2* with T2D, insulin resistance assessed by lnHOMA-IR, or MetS.

This relatively large study confirms that *ADIPOQ* tSNPs are significantly associated with serum adiponectin. We found the minor alleles at rs12637534, rs1648707/rs6810075 and rs17366568 to be associated with lower serum adiponectin concentration (*P*<1×10^-5^), while the minor alleles at rs10937273, rs16861209/rs822387, rs822395, rs3774261, rs6444175 and rs17373414 were associated with higher levels (*P*≤0.02). These associations and their direction are consistent with the hypothesis that circulating adiponectin levels are in part determined by genetic influences [3-7,9,10,29-31].

We failed to replicate previous findings in the literature for *ADIPOQ* SNPs rs2241766, rs1063537 and rs3821799 that have been reported in two large GWAS [3,31], but not supported by other studies [4,8]. In addition, there were three tSNPs (rs266729, rs62625753 and rs17366743) reported as associated with serum adiponectin that did not show a significant association with adiponectin levels in the present study. LD data available from Heid et al. [3] show that rs266729 is correlated (r^2^>0.60, D’>0.80) with tSNPs which were significant in our study (rs6810075, rs182052 and rs1648707).

The lack of replication of the association between adiponectin levels and SNPs previously identified in GWAS is intriguing. As several rare population-specific coding variants have been reported to be strongly associated with adiponectin level [6,32,33] One possible explanation for the observed discrepancies may be due to synthetic associations i.e. the fact that population-specific rare variants are in partial linkage disequilibrium with common variants [34].

In the present study, the significantly associated tSNPs were located across all regions of the gene, including the promoter, exonic, intronic and 3^′^-untranslated regions. Interestingly, all of the tSNPs downstream of exon 2 were associated with an increased serum adiponectin.

We observed no association between *ADIPOR1/R2* variants and serum adiponectin which is consistent with most other studies [14,35-37]. Recent genome-wide studies have identified variants in a third adiponectin receptor, T-cadherin, that affect circulating adiponectin [2,4,38,39]. T-cadherin, encoded by *CDH13,* is a receptor for hexameric and high-molecular-weight adiponectin, but not for the trimeric or globular species as measured in the present study. Nevertheless, variants in *CDH13* warrant further investigation.

Despite the fact that *ADIPOQ* variants are associated with a modest amount of variance in total serum adiponectin (<5%), interventions aimed at the encoded protein may affect secreted adiponectin to a much greater extent. Indeed, the thiazolidinediones have been shown to increase circulating adiponectin 2–2.5 fold in patients with diabetes, although the exact mechanism of action remains unknown. Other population studies have reported similar quantitatively small effects of *ADIPOQ* SNPs, and show that there are a large number of SNPs contributing independently to the variation [3,7,40]. A recent large multiethnic GWAS identified ten new loci, in addition to *ADIPOQ* and *CDH13,* that affect circulating adiponectin, but the combined multi-SNP genotypic risk score only explained 5% of the variance in serum adiponectin [31]. The high heritability estimates for adiponectin may be explained by as-yet undiscovered rare variants with strong effects, additional unknown common loci with small effects, epigenetic mechanisms, or by copy number variations (CNVs). *ADIPOQ* contains a high number of CNVs and the effect of these on circulating adiponectin is unknown. Complete sequencing of the *ADIPOQ* locus has been performed in a large population where seven SNPs were shown to influence adiponectin levels but the authors found no evidence of an association between these SNPs and T2D in a diabetic case control study [33].

Recently, studies have identified rare population-specific mutations that account for 17% of the variance in serum adiponectin [32,41]. Further analysis of gene-gene and gene-environment interactions may explain the remaining heritability.

Despite putative effects of the adiponectin receptors on tissue adiponectin activity, we found no evidence that tSNPs in *ADIPOQ*, or *ADIPOR1*/*R2* were associated with either insulin resistance, or MetS. This supports the findings of two other studies [5,33]. However, we did find a significant association between the allele score of the top nine SNPs and T2D in the case–control study without adjusting for adiponectin levels. Multi-SNP genotypic risk score for adiponectin-decreasing alleles has been associated with BMI, WHR, fasting insulin, HOMA-IR, 2-hour post OGTT glucose, T2D, triglycerides and HDL-cholesterol [31]. In the current study a post-hoc analysis revealed that adjusting for adiponectin levels strengthened the association with 3 independent SNPs, rs12637534, rs16861209, rs17366568 and T2D. The strengthening of this association provides strong evidence that the genetic determinants of adiponectin levels are shared with T2D using a Mendelian randomisation approach [13].

There have been numerous studies investigating the association of adiponectin gene and related polymorphisms with T2D. Two recent large meta-analyses showed that the G vs C allele of rs266729 might be a risk factor for T2D [42,43], while the rs17300539 A allele was shown to be a risk factor only in European Caucasians [43]. The latter SNP is in complete LD with rs16861209 that we have identified in our Caucasian populations.

The limitations of this study include the use of a single baseline serum adiponectin measurement which may not fully characterise long-term adiponectin exposure and effects. Nevertheless, other prospective studies that analysed repeated serum adiponectin measurements, on average one year apart, reported high intra-class correlations above 0.71 [35,44], indicating high reproducibility of serum adiponectin levels. In the present study, total serum adiponectin levels were measured, whereas recent evidence suggests that the high molecular-weight form of adiponectin, rather than the low molecular-weight forms, may be the bioactive protein [45].

## Conclusions

In summary, we have comprehensively analysed the genetic variants and haplotypes of *ADIPOQ* and its two receptor genes using a tagSNP approach in two general populations and a diabetic cohort, in total comprising 3,322 well phenotyped subjects. We found that age, gender and BMI accounted for approximately one-third of the variation in serum adiponectin in the general population cohorts and just over one-fifth in a diabetic population. Variants in the *ADIPOQ* gene accounted for <5% of the variation in serum adiponectin and genetic variants in *ADIPOQ* and its receptors does not appear to contribute to the risk of insulin resistance or metabolic syndrome. However, a multi-SNP genotypic risk score for *ADIPOQ* did associate with T2D independent of adiponectin levels. The factors that underlie the majority of the variation in serum adiponectin remain unknown, but given the potential beneficial effects of this adipokine, merit further study.

## Abbreviations

MetS: Metabolic syndrome; T2D: Type 2 diabetes; GWAS: Genome-wide association study; SNP: Single nucleotide polymorphism; tSNP: Tag single nucleotide polymorphism; BHS: Busselton Population Health Survey; CUDAS: Carotid Ultrasound Disease Assessment Study; FDS: Fremantle Diabetes Study; IR: Insulin resistance; lnHOMA-IR: Homeostatic model of assessment; BMI: Body mass index; NCEP: National Cholesterol Education Program; HWE: Hardy-Weinberg equilibrium; LD: Linkage disequilibrium.

## Competing interests

The authors declare that they have no competing interests.

## Authors’ contributions

KEP drafted the manuscript and assisted in the collection, analysis, and interpretation of the data. GC, NMW, and SW carried out the analysis and interpretation of the data. JB and JH coordinated the data collection and interpretation of results. WD was responsible for the FDS data collection and validation. TD is the principal investigator of the FDS. All authors participated in the study design and coordination. All authors read and contributed to the final version of the manuscript.

## Pre-publication history

The pre-publication history for this paper can be accessed here:

http://www.biomedcentral.com/1471-2350/14/15/prepub

## Supplementary Material

Additional file 1 **Details of the SNPs genotyped in this study.** The location and minor allele frequency (from HapMap) of each tSNP are given, together with the allele frequency and genotype distribution in each cohort. Click here for file

Additional file 2 **Association of *ADIPOQ *SNPs with serum adiponectin for each cohort.** The marginal geometric mean values of adiponectin according to genotype at significant tSNPs are shown by individual cohort. Data are estimated marginal mean (MM) serum adiponectin mg/L (95% CI) after adjusting for age, gender and BMI. *P*-values were taken from the likelihood ratio test: model without genetic covariates vs model with genetic covariates. Q-values were calculated in each cohort using all 21 *ADIPOQ* tSNPs. *, †, ¥ represents tSNPs that are in LD in our study. Click here for file

Additional file 3 **Details of the additional SNPs captured by our tagSNP approach.** Haploview was used to determine additional SNPs that were captured by the tSNPs genotyped in the present study (*r*^2^≥0.80, MAF>0.01). This analysis was not applicable to tSNPs rs860291, rs822395 and rs2241766 as they are not listed in HapMap. * tSNP associated with serum adiponectin in our cohort. Click here for file
